# Journey of candidates who were unmatched in the Canadian Residency Matching Service (CaRMS): A phenomenological study

**DOI:** 10.36834/cmej.69318

**Published:** 2020-07-15

**Authors:** Basia Okoniewska, Malika A. Ladha, Irene W. Y. Ma

**Affiliations:** 1 Department of Family Medicine, University of Calgary, Cumming School of Medicine, Alberta, Canada; 2 Division of Dermatology, Department of Medicine, University of Calgary, Cumming School of Medicine, Alberta, Canada; 3 Division of General Internal Medicine, Department of Medicine, University of Calgary, Cumming School of Medicine, Alberta, Canada; 4 W21C, University of Calgary, Alberta, Canada

## Abstract

**Background:**

Each year, a number of medical students are unmatched in the Canadian Residency Matching Service (CaRMs) match. Blog posts from previous unmatched students suggest that being unmatched is associated with significant stress. However, no studies have explored the collective experiences of candidates who are unmatched. This study seeks to explore the experiences of Canadian students who were unmatched in the first iteration of their CaRMS applications.

**Methods:**

This was an interview-based qualitative study using a phenomenology approach to explore the perspectives of 15 Canadian participants from seven universities who had previously experienced being unmatched between 2011 and 2017 in CaRMS. Telephone interviews were conducted using a semi-structured guide focusing on the experiences in the following domains: the overall unmatched experience; perceived reasons leading to their unmatched status; resources employed; barriers experienced; recommendations; and, their eventual career outcomes. Field notes were analyzed independently by all authors using thematic analysis and authors independently identified major themes. To reconcile divergent impressions and better situate qualitative impressions of our participants, we used publicly available quantitative data from CaRMS to calculate relevant odds ratios.

**Results:**

Our participants universally reported negative emotions, concerns regarding privacy and confidentiality breaches, and stigma faced. Systemic challenges faced by our participants included: lack of information, pressures perceived from undergraduate medical education to apply in the second iteration to specialties that they did not want, and logistical issues such as financial challenges, licensing and scheduling issues. The utility of peer support differed for individual participants, but all those who had support from other unmatched candidates felt that to be useful.

**Conclusions:**

Our participants reported significant challenges faced after being unmatched. Based on these experiences, we identified key areas of support needed for candidates through their unmatched journey.

## Abstract (fr)

**Contexte:**

Chaque année, un certain nombre d’étudiants en médecine ne sont pas jumelés par le Service canadien de jumelage des résidents (CaRMs). Selon les billets de blogue publiés par d’anciens étudiants non jumelés, le fait de ne pas être jumelé occasionne un stress considérable. Cependant, aucune étude n’a exploré l’expérience globale des candidats non jumelés. La présente étude a pour but d’examiner les expériences des étudiants canadiens qui n’ont pas été jumelés lors du premier tour du jumelage de leur demande au CaRMS.

**Méthodes:**

Pour mener cette étude qualitative basée sur des entrevues, nous nous sommes servis d’une approche phénoménologique afin d’explorer les perspectives de quinze (15) participants canadiens de sept universités différentes qui n’ont pas été jumelés par le CaRMS entre 2011 et 2017. Lesentrevues téléphoniques ont été réalisées suivant un guide semi-structuré ciblant les expériences dans les domaines suivants : l’expérience globale de non-jumelage; les raisons perçues menant au non-jumelage; les ressources utilisées; les obstacles rencontrés; les recommandations et les résultats éventuels sur leur carrière. Les feuilles de route ont été analysées de manière indépendante par tous les auteurs en utilisant l’analyse thématique et les auteurs on indépendamment identifié trois thèmes majeurs. Afin de concorder les impressions divergentes et de mieux situer les impressions qualitatives de nos participants, nous avons utilisé les données quantitatives du CaRMS mises à la disposition du public pour calculer les rapports des cotes pertinents.

**Résultats:**

Nos participants ont tous rapporté des émotions négatives, des inquiétudes en ce qui concerne la violation de leur vie privée et de la confidentialité ainsi que de la stigmatisation. Parmi les défis liés au systèmeauxquels nos participants ont fait face,’on compte : le manque d’information, la pression perçue lors des études pré-graduées en médecine de faire une demande de spécialité non désirée lors du second tour du jumelage et les problèmes de logistique comme les difficultés financières, l’obtention d’un permis et les conflits d’horaire. Le degré d’utilité du soutien par les pairs a été différent pour chaque participant, mais tous ceux qui ont reçu de l’appui d’autres candidats non jumelés ont estimé qu’il avait été utile.

**Conclusions:**

Nos participants ont signalé qu’ils ont dû faire face à d’importantes difficultés après avoir été non jumelés. En nous basant sur ces expériences, nous avons identifié les principaux types de soutien qui devraient être fournis tout au long du parcours des candidats non jumelés.

## Introduction

Final-year medical students in Canada undergo an application process to obtain a training position within a post-graduate residency program. This process, colloquially known as “the match,” is completed using the Canadian Residency Matching Service (CaRMs), a national not-for-profit, fee-for-service organization (www.carms.ca). Students apply to individual residency programs across Canada through CaRMs, which then distributes the applications to the residency programs. Residency programs then review the applications and invite select candidates for interviews. Both the candidate and program submit rank lists based on their desired programs or candidates, respectively. This process culminates in match day: applicants log onto the CaRMS system to find out which city and specialty to which they have matched. The result is legally binding; thus, a candidate does not have the legal right to withdraw from the match or residency program.

Each year, a number of Canadian medical students are unmatched in the first iteration of CaRMS. By not being able to secure a residency position in Canada, unmatched students face a future in which they will not be able to practice medicine. On the surface, the consequence of not matching in the first iteration of CaRMs may not appear significant. After all, applicants have the choice to enter the second iteration, where candidates can apply to unfilled positions across Canada, or enter the match in the following year. However, even for those who are successful in matching in future iterations, they may not match to their choice of specialty or geographic location. For some, this results in a career practicing in a specialty in which they have limited interest. For others, this results in an unplanned and unwelcomed move away from their families and support network. Furthermore, being unmatched may result in not only an unclear professional future and its associated stigma, but also potentially serious financial and emotional consequences; some are so distraught that they instead choose even suicide.^1^

In 2018, the number of unmatched students reached a record high of 169 students (7.6%) followed by 5.9% of students unmatched in 2019.^2,3^ Strategies to minimize the risk of becoming unmatched have not been effective. Over the last 10 years, students have been steadily increasing the number of programs to which they apply, from a mean of 10.8 programs in 2009 to now 21.2 programs per applicant in 2019.^4^ There is mounting pressures on medical students to choose a specialty early on in their undergraduate medical training;^5^ candidates have to balance the benefits of acquiring broad-based skills and knowledge with the harms of appearing unfocused to residency selection committees.^6^ Aside from some broad recommendations from the Canadian Federation of Medical Students, such as providing better education about red flags to students and increasing the number of residency spots,^7^ it is unclear how best to prevent ending up unmatched.

Being unmatched is a profound life experience full of stress, uncertainty and multiple challenges.^1,8,9^ There are only a few blog posts from unmatched candidates describing their own experiences,^9-12^ For example, Dunkley speaks of a lack of a support system and feeling so alone after being unmatched,^9^ while Smith recounts her reaction to being unmatched of shock, grief, and devastation,^11^ sentiments echoed by Persad.^12^ In addition, there is limited understanding of the factors which may lead a student to be unmatched; selection criteria were felt to be vague and subjective.^10^ We found no literature that describes or examines the phenomenon of being unmatched: its impact, consequences, and collective lived experiences. The purpose of this study was to uncover areas for improvement on how to support unmatched candidates by investigating the phenomenon of being unmatched.

## Methods

This was an interview-based qualitative study using a phenomenology approach to explore the perspectives of participants who had previously experienced being unmatched in CaRMS.^13^ We chose a phenomenology approach because based on the experiences described in the literature,^9-11^ we hypothesized that unmatched candidates’ lived experiences may share some common themes and that their collective lived experiences can be better captured. In better describing this phenomenon, we hope to help future unmatched candidates through their journey. The study’s findings are reported based on the Standards for Reporting Qualitative Research.^14^ Ethics approval for this study was obtained from the Conjoint Health Research Ethics Board (REB17-1676).

### Participants and recruitment

Participants included were those who 1) had been unmatched in their first iteration of the CaRMS match between 2011 and 2017, 2) consented to share their experiences in this study, and 3) were able to participate in the interview.^15^ We identified 15 participants from across seven medical schools in Canada by snowball sampling technique.^16^ The initial group of potential participants (*n* = 10) were those who met the above inclusion criteria and were known to the resident researchers on our team. Five additional consenting participants were then identified by the snowball technique. To maintain confidentiality, the faculty preceptor (IM) involved in this study is blinded to the identity of the participants and is not involved in either the recruitment or the interview processes.

Telephone-based interviews were conducted by trained resident researchers (BO and ML) between February and September 2018 using a semi-structured interview guide (Appendix 1). Interviews were conducted on the phone and lasted between 30 minutes to one hour. Because of the potential sensitive nature of the topic, the interviews were not recorded; but anonymized field notes were compiled by the resident researchers during the interviews.

The questions during the interview explored the following: the participant's’ overall unmatched experiences; circumstances the participants believed led to their unmatched status; resources employed; barriers experienced; recommendations; and, their eventual career outcomes.

Upon completion of the interviews, field notes were analyzed independently by all authors using thematic analysis. Because our interview questions dealt not only with individual experiences of the phenomenon of being unmatched, but also more broadly issues such as systems support, finances, processes, and resources, we chose to conduct thematic analysis because of its flexibility.^17^ By reading and re-reading field notes from each interview multiple times, all research members independently and inductively identified major themes. The research team met in-person monthly over four months to debrief and discuss discrepancies and disagreements in coding. Discordances were resolved by discussion and further refinement of the coding structure, followed by recoding of the data.

Lastly, to reconcile qualitative impressions of participants regarding school factors that resulted in the unmatched status,^18^ where applicable, we evaluated quantitative data publicly available from CaRMS to calculate relevant odds ratios, to better situate our participants’ perspectives.^18,19^

## Results

Table 1 shows the demographic data of the participants.

**Table 1:** Baseline demographic of the 15 participants

Characteristic	N (%) or mean ± standard deviation (SD)
Gender	
Male	4 (27%)
Female	11 (73%)
Level of Education	
No prior degree	3 (20%)
Prior bachelor’s degree	9 (60%)
Prior master’s degree	2 (13%)
Prior PhD degree	1 (6%)
Mean no. of specialties applied	1.5 ± SD 0.7
Mean no. of locations applied	10.6 ± SD 5.6
Mean no. interviews offered	5.9 ± 3.3
Mean no. of interviews completed	5.5 ± 3.0
Mean no. of interviews ranked	7.1 ± 6.3 (range 1-28)
Post-Unmatched Outcome	
Extension of clerkship	5 (33%)
Graduation from medical school	3 (20%)
Further training (postgraduate degree/fellowship)	3 (20%)
Second iteration CaRMS match	4 (27%)

We discussed six major areas with the participants: personal challenges, perceived circumstances that contributed to participants not matching, systemic challenges, resources available, overall outcomes of not matching, and suggestions for future unmatched candidates.

### Personal challenges

In discussing challenges faced by the participants during the unmatched process, three key themes emerged which were universally felt by all 15 of our participants: negative emotions, concerns regarding privacy and confidentiality breaches, and stigma faced by the participants.

Negative emotions emerged as one of the most significant and consistent themes that candidates used to describe their overall experiences. These emotional experiences ranged from disbelief, grief, self-doubt, shame, isolation and bitterness. Many candidates described ranges and lability of their emotions as an “emotional rollercoaster”:

*I was deeply hurt from going unmatched: the absolutely numbing and excruciating pain that sporadically, though regularly pierces, rips through and transcends every dimension of my person is beyond describable. It is additionally sprinkled with intense bouts of utter confusion, of social humiliation, of disappointment, of isolation, of uncertainty, of betrayal, of shame, of self-questioning. It is such a wrenching experience that as much as I am very thankful for the lifetime’s worth of character building it has served me, I sincerely do not wish it upon anyone*.

Participants also described concerns for their own mental health, although only three labelled their states as “depression.” They also described a sense of vulnerability and commented on the difficulty in watching as their classmates move on: “*At one point, they had all of my class stand up and celebrate the new graduates and new MDs. I remember not knowing what to do, so I just kept sitting. I felt left behind*.”

Many of the participants felt mentally unprepared for being unmatched, despite cognitively recognizing its possibility: “*I knew the possibility of going unmatched was there, but there was no part of me that thought I would go unmatched*.” For many of the participants, being unmatched was the first and biggest professional setback the participant had experienced to date.

Other themes that emerged included the need for privacy and fear of confidentiality breaches. Although some participants were open with their peers about their unmatched status, a number of candidates wished to hide their unmatched status from their preceptors, other programs, and peers. This is well expressed by one of the participants “*I tended not to tell any residents I worked with, just because I didn’t know what the reaction would be. I would skirt around certain questions."* Furthermore, most candidates reported that their unmatched status was shared amongst their medical school class within days, effectively breaching confidentiality.

Stigma was another key theme that emerged within this category. Participants either described experiencing stigma from colleagues (medical students, residents) or from preceptors or programs, as expressed by this participant: *Everyone automatically assumes that if you go unmatched, you are inadequate and it was something you did*.” Another participant linked such stigma to shame, “*I didn’t tell people in my electives, because I thought it would put me at a disadvantage. I felt ashamed*.” This stigmatization was not, however, experienced by all participants, although the concern for its possible presence was still felt. For example, one participant stated: *“I appreciated no actual stigma, although I feared it from all.”*

The above themes may be inter-related. For example, one comment from a participant suggested that stigma and the need for privacy may be related: “*I didn’t always share that I was unmatched because I was worried that people would go looking for a reason that I didn’t match*.”

### Perceived circumstances for not matching

Participants felt that being unmatched related to:

1. The matching process. Comments about the matching process suggest that the participants felt they had little control over the outcome. Two participants felt that being unmatched was an inevitable result of a simple supply and demand issue: “*It’s like a game of musical chairs. You go around and around and then all the chairs are gone. There is not enough spots for everyone*.” The subjectivity as well as the fairness of the matching process itself was also raised: “*What I didn’t understand was that things in CaRMS is not about merit. It’s about your connections. It’s about networking*.”
2. School factors. Four participants commented on school-related factors as a possible contributor (e.g. three-year program, insufficient elective time exposure). However, review of five years of CaRMS data (2015 to 2019, inclusive), suggests that the odds of matching for a 4-year school was not different than that of a 3-year school (odds ratio 0.89, 95% confidence interval 0.73 to 1.08, *P* = 0.23).
3. Match strategy: Five participants felt that being unmatched was related to ranking too few programs, although in at least two of these cases, family circumstances did not allow sufficient mobility to do so.

### Systemic challenges

Participants experienced a number of commonly identified systemic challenges when they went unmatched. Lack of information was a key theme that emerged, and this stemmed from three domains: lack of feedback from programs, lack of clarity and transparency regarding the unmatched process, and lack of information from undergraduate medical education (UME) departments. With respect to lack of feedback from programs, the majority of participants attempted to contact the programs they applied to asking for constructive feedback. They received either no response or a generic response. For example,

*I emailed all the programs I applied to and asked for feedback. It was really helpful, but it’s a bit of a black box, and sometimes candidates are left scrambling. Being now on the other side, I know it’s ugly and it’s political. It’s competitive. Program directors have their hands tied and can’t really give out that political ugliness, they can’t shed light on the process. So you usually get vague things back*.

Only one participant described having program directors responding to their request in a favorable manner, stating they were well liked but unfortunately the program was very competitive and had a small number of spaces available. Secondly, there was a lack of clarity regarding the process after being unmatched; participants felt it was unclear what it meant for the unmatched candidate:

*I think people need to talk about what happens and what the process is when you go unmatched so it is not a mystery. This process needs to be transparent. Trying to figure out what you can and can’t do after the fact is very difficult, and you are not in the right mind set to do it*.

Lastly, information from UME was also lacking. For example,

*Extension of clerkship was only offered. They did not offer me anything else up front. I asked them what would happen if I chose to graduate, and I had to really dig this out of the UME. They eventually told me that if I chose to graduate, the medical school would offer no further support*.

Six participants reported experiencing pressure from UME to apply in the second iteration to specialties that they did not want:

*The medical school stressed that I needed to apply to the second iteration, and to apply to all the programs. I was told to broaden your horizon. That was really frustrating to hear, especially right after not matching. It was like they were pushing me into something to just get their match results up. At the time, I still very much wanted that one specialty*.

Another participant stated:

*They were not tailoring it to what was best to the individual. It was more of pushing you to match, than to actually sit down and dig into what you wanted to do. There was no talk about what your career ambitions are, what would be a good fit, what your life goals were to be*.

Participants encountered logistical issues such as financial challenges, licensing and scheduling issues:

*…they would not extend my line of credit (LOC) because I’m not affiliated with a medical school or residency program. I realized I was financially screwed. I asked the bank to up my LOC, they said no. They froze my LOC since I was no longer a student. It really hit me hard and low. It was just another reminder that you are lost in society. It felt like I had a criminal record*.

Candidates who chose to graduate but still wanted to do electives or maintain clinical experience described difficulties obtaining clinical trainee licenses or insurance: “*Setting up a clinical trainee license was very difficult. No institutions would cover you anymore since you are not affiliated with a medical school, so I had to find a physician that could vouch for me.”* Because of these barriers, another participant did not even attempt to attain a license: “*I had no insurance and was not affiliated with any medical school since I had graduated.”*

Lastly, with respect to scheduling issues, participants indicated that they were given no further time to attend second iteration interviews. Some felt significant time pressures, stating there is a very quick turn-around time for the second iteration applications, leaving candidates scrambling to write personal letters and asking for new reference letters while still performing clinical duties and studying for exams. Participants who chose to extend often had difficulty booking electives, as many electives required a minimum of 8-10 weeks’ notice, and competitive electives were often booked months in advance.

### Resources available to unmatched candidates

All candidates were contacted by the UME shortly after not matching (i.e. one to two days) and spoke with someone at UME (e.g. career counselors, assistant deans). However, the majority of participants felt that UME did not provide adequate support, information, options, or leadership. Only one participant (and this was unique to this participant) had a particularly good experience with UME:

*The calling the day before was hugely helpful, just having the day to adjust. [UME was] very supportive, meeting with me whenever I wanted, offering to review my applications, giving me as much time off as I needed (for mental health or to go to interviews). [UME had a process] in place to make it easy for me to defer graduation. They had an idea of how to go forward*.

The utility of other resources differed for individual participants. Some found colleagues in medicine to be helpful and supportive, but many found they did not know how to act: “*I wish there was some way of sheltering people from the constant questions of “where did you match to?*” Some found friends outside of medicine helpful: “*People in medicine see the culture of CaRMS in a different way than people outside of medicine. Within medicine it was a catastrophic response. People outside were much more reasonable. It made me realize CaRMS is not the be-all-end-all*.” Others expressed frustration that non-medicine friends did not fully understand processes/culture. Ten participants connected with prior unmatched candidates. Support from prior unmatched candidates were universally felt to be useful: “*They acted as a huge support for me. They knew the exact emotions I was feeling and helped normalize and guide me through it all*.”

### Overall outcomes of not matching

By and large, most participants felt content with the final outcome. Ten participants ultimately matched to a different specialty. Among these participants, two expressed reservations regarding their ultimate specialty (lack of belonging/lack of competency) and one experienced difficulty transferring out of the program, resulting in simply giving up and staying in the current program. All candidates indicated that through this unmatched process, they experienced growth either personally or clinically.

This was well expressed in the following quote:

*I think it was a very positive thing, being unmatched, but it’s the product of hindsight. I think if I did something else, I would still look back at this as a very positive thing. It’s a big a failure (although I don’t like saying it’s a failure). Probably, the better phrase is to say it was a big setback, and most people don’t experience it*.

### Suggestions for future unmatched candidates

There was consensus that provision of information regarding what happens when a candidate goes unmatched, and information on options and resources available at their current institution would be helpful for unmatched candidates. Table 2 shows potential options for unmatched students, collated based on information offered by our participants. Importantly, candidates stressed that this information needs to be given prior to the CaRMS match. Our participants felt that a culture change is needed: the current “no-fail” culture within the medical profession needs to change to remove stigma associated with being unmatched: “*It’s kind of like mental health – no one likes to talk about it, but we should be able to talk about it without judgment.”*

**Table 2:** Summary of advantages and disadvantages of options available to candidates who are unmatched after the first iteration of the Canadian Resident Matching Service (CaRMS), as articulated by our 15 participants

	Advantages	Disadvantages
**Extension of Clerkship** **(i.e. apply to CaRMS in the first iteration of the following cycle)**	Ability to complete electives in any field and at other sites	No guarantee for a successful match in the first iteration of the following cycle of CaRMS
	Opportunity to strengthen future CaRMS applications by visiting more sites, expanding areas of interest, and securing stronger letters of references	Financial burden – required to pay full tuition and extension of line of credit is not guaranteed
	Opportunity to strengthen clinical skills	Will not graduate with candidate’s current class, thus may experience difficulty witnessing colleagues move on to the next stage of training
	Opportunity to expand research portfolio	Difficulties in securing electives
	Ability to reapply to original specialty and to increase competitiveness for other specialties through clinical electives	Burden of stigma on clinical rotations
**Graduation from Medical School**	Ability to reapply to original specialty	Support from undergraduate medical education (UME) is discontinued (no contact or support can be provided)
	Graduate with candidate’s current class	Financial instability – no secured source of income and extension of line of credit is not guaranteed
	Ability to engage in activities outside of medicine (i.e. travel, non-medical careers)	Burden of stigma
	Opportunity to expand research portfolio	Difficulty in securing clinical insurance for clinical electives; lack of clinical exposure can negatively impact future CaRMS applications
**Further Training (post-graduate degree or fellowship)**	Ability to reapply to original specialty	Deadlines for many graduate programs are earlier than CaRMS deadlines
	Opportunity to expand research portfolio; this option provides the most dedicated time for research	Support from UME is discontinued (no contact or support can be provided)*
	Some fellowships are paid, offering financial compensation	Potential for financial instability, especially if tuition is required
	Graduate with candidate’s current medical class	Difficulty in securing clinical insurance for clinical electives; lack of clinical exposure can negatively impact future CaRMS applications
**Second Iteration CaRMS Match**	Graduate with candidate’s current medical class	Fear of not liking ultimate specialty
	Financial compensation guaranteed	Perception of being a “second tier” resident
		Feeling indebted to program/institution
		Difficulty watching colleges begin their residency of choice
		Subjective fear of retaliation
		Fear of stigma

* Exception: support from UME can be maintained if students do not graduate from medical school and complete the extra degree in conjunction with their medical degree

## Discussion

Collective experiences of unmatched candidates in the first iteration of their CaRMS match had not been previously described and explored; there have been a few prior blog posts from individuals about their personal unmatched experiences,^9-12^ and a recent narrative analysis.^20^ Our results suggest that while individual circumstances and outcomes differed amongst candidates who went unmatched in the first iteration of their CaRMS match, universally, they described an uphill battle of isolation, stigma, grief, and uncertainty. Some of these sentiments were also shared in the published personal experiences of Smith and Dunkley,^9,11^ while others described shame that was echoed by some of our participants.^20^ Our study participants further shared how these emotions contributed to their sense of isolation and loneliness. The universal experience of grief, stress, and emotional turmoil that the participants described and shared in this study, under no uncertain terms, should raise alarm bells to medical educators and point to the urgent need of support for unmatched candidates.

Based on the candidates’ collective experiences, support will need to come in several forms. First and foremost, mental health support is needed and should be provided in a timely and longitudinal fashion. This support needs to be holistic and accessible. A mentorship network opportunity involving previously unmatched residents who are willing to mentor unmatched candidates locally should be made available. Students should be connected to the existing Canadian Federation of Medical Students Unmatched Peer Mentorship Network.^21^ Second, access to information should be provided. This includes available options for each school, advantages/disadvantages of each of those options, steps, timelines and contact information for each option. Information regarding costs/financial planning, insurances, and licensing requirements should be given. These options should be freely pursued by students and not be coerced. Third, support for processes should be in place. Our participants felt woefully unprepared for navigating the unmatched process and scheduling. Support needs to be readily available: time off of clinical duties for interviews and assistance for urgent elective scheduling should be offered. Last, there needs to be an underlining culture shift within the medical community regarding being unmatched in the CaRMS process. Normalization of this process by education, role modeling, and healthy conversations amongst learners, educators, and administrators are needed to reduce the stigma. Every year, students go unmatched. Yet despite this, it was surprising to hear how many participants struggled to get information and how challenging it was for them to navigate the system. Preventative measures should be in place for these predictable events that occur yearly.

There are a few limitations of this study. First, due to the sensitivity of the topic, it was difficult to recruit participants, resulting in a small sample size. Nonetheless, our 15 participants’ described lived experiences demonstrated remarkable consistency in their negative emotions and challenges faced after being unmatched. Second, our study is subject to recall bias. Third, while the majority of the participants ultimately did match, we did not consistently capture additional information about their outcomes, such as when they matched. Additional studies on unmatched participants’ outcomes would be helpful. Fourth, although whenever possible, the interview was done by an interviewer not known to the participant, a number of these participants were ultimately interviewed by a researcher known to them. Although our team met monthly to debrief, our schedules did not permit more frequent debriefing to discuss reflexivity and how the interviewers’ personal experiences and inherent biases may have affected the interviews.^22^ Finally, due to the sensitive nature of the topic, we opted to rely on field notes rather than transcripts from recordings of the interviews. The use of field notes, however, may have resulted in a loss of information and details; interpretations of the field notes may also have resulted in a biased and/or over-simplistic interpretation of the data.^23^ Yet despite these limitations, the collective experiences reported by our participants are noteworthy and should not be ignored by educators.

## Conclusions

This qualitative research study explored unmatched CaRMs candidates’ experiences and the emotional and systemic challenges they faced. Based on our data, we have identified key areas of support needed for candidates as they journey through their unmatched status.
